# Historical account of the national health insurance formulation in Kenya: experiences from the past decade

**DOI:** 10.1186/s12913-015-0692-8

**Published:** 2015-02-12

**Authors:** Timothy Abuya, Thomas Maina, Jane Chuma

**Affiliations:** Population Council, P.O Box 17643–00500, Nairobi, Kenya; Futures Group, Health Policy Project, P.O Box 3170–00100, Nairobi, Kenya; Ministry of Health, P.O. Box: 30016–00100, Nairobi, Kenya; KEMRI-Wellcome Trust Research Programme, P.O Box 230–80108, Kilifi, Kenya; Centre for Tropical Medicine, Nuffield Department of Clinical Medicine, University of Oxford, Oxford, UK; School of Economics, University of Nairobi, P. O. Box 30197–00100, Nairobi, Kenya

**Keywords:** Health insurance, Universal health care coverage, Policy Analysis

## Abstract

**Background:**

Many Low-and-Middle-Income countries are considering reviewing their health financing systems to meet the principles of Universal Health Coverage (UHC). One financing mechanism, which has dominated UHC reforms, is the development of health insurance schemes. We trace the historical development of the National Health Insurance (NHI) policy, illuminate stakeholders’ perceptions on the design to inform future development of health financing policies in Kenya.

**Methods:**

We conducted a retrospective policy analysis of the development of a NHI policy in Kenya using data from document reviews and seven in depth interviews with key stakeholders involved in the NHI design. Analysis was conducted using a thematic framework.

**Results:**

The design of a NHI scheme was marked by complex interaction of the actor’s understanding of the design, proposed implementation strategies and the covert opposition of the reform due to several reasons. First, actor’s perception of the cost of the NHI design and its implication to the economy generated opposition. This was due to inadequate communication strategies to articulate the policy, leading to a vacuum of factual information flow to various players. Secondly, perceived fear of implications of the changes among private sector players threatened support and success gained. Thirdly, underlying mistrust associated with perceived lack of government’s commitment towards transparency and good governance affected active engagement of all key players dampening the spirit of collective bargain breeding opposition. Finally, some international actors perceived a clash of their role and that of international programs based on vertical approaches that were inherent in the health system.

**Conclusion:**

The thrust towards UHC using NHI schemes should not only focus on the design of a viable NHI package but should also involve stakeholder engagements, devise ways of improving the health care system, enhance transparency and develop adequate governance structures to institutions mandated to provide leadership in the reform process to overcome covert opposition.

## Background

Universal Health Coverage (UHC) currently dominates the global policy agenda. Governments and policy makers are considering how to review their health financing systems to make progress to UHC. The 58th World Health Assembly urged member states to ensure that “health-financing systems introduce prepayment mechanisms for the health sector, with a view to sharing risk, avoid catastrophic health-care expenditure and impoverishment of individuals as a result of seeking care” [1]. The 2010 World Health Report identified the important role of health financing in achieving universal health coverage (UHC) [2], while the 2013 WHO report was dedicated to UHC, the idea is being considered as one of the health system goals post 2015, when the millennium development goals come to an end.

Health care financing has three interrelated functions namely: revenue collection, pooling and purchasing [3]. Revenue collection is the process by which health systems receive money from households and organizations. Pooling is the accumulation and management of revenues to ensure that the risk of paying for health care is borne by all the members of the pool and not by individual contributor [4,5]. Purchasing is the process by which pooled funds are paid to providers in order to deliver a set of health interventions on behalf of the population for which the funds are pooled [4]. Recent policy discussions have focused on how to restructure these functions to ensure that health systems in Low-and-Middle-Income Countries (LMICs) are primarily funded through prepayment mechanisms that allow for risk pooling and income cross-subsidization.

Health systems in many LMICs have been primarily funded through out-of-pocket (OOP) payments. OOP payments are a major barrier to access; they promote inequities and contribute towards household poverty [6-10]. These concerns have led to a shift in policy debates, away from OOP payments as the main sources of health care funds, towards prepayment mechanisms, including tax funding and/or health insurance contributions [11]. Health insurance has gained popularity over tax funding, particularly in Africa because tax driven health systems in developing countries face challenges of a small formal sector, low institutional capacity to collect taxes and a lack of tax compliance. Health insurance schemes, it is argued, have fewer difficulties in identifying their members, in collecting their contributions and their benefits and are said to be more visible and linked to contributions. Health insurance protects individuals incurring high costs at the time of illness, thereby promoting access to health care, particularly in settings where the government subsidizes premiums for the poorest population. Consequently, health insurance is potentially being viewed as a mechanism for overcoming existing structural inequities in Africa [1,12,13].

Kenya is one of the few African countries that have had a national hospital insurance scheme in existence since the 1960s. Membership to the National Hospital Insurance Fund (NHIF) is mandatory for all Kenyans in formal employment and voluntary for those in the informal sector. The NHIF has been criticized for poor quality of care in accredited facilities, a cumbersome claiming process and location of offices in urban areas where the minority of the population live [14]. To address some of these concerns, the country is currently considering introducing a National Health Insurance Scheme (NHIS), which will include transforming the NHIF to cover all Kenyans, for both outpatient and inpatient services irrespective of their ability to pay. Under the new arrangement, the NHIF will be reformed and will purchase comprehensive health insurance for all Kenyans.

The NHIS proposal, developed in 2004/2005 drew attention both locally and internationally but was met with resistance from various stakeholders. The NHIS Bill was highly controversial; it was nevertheless passed in parliament, but the president declined to sign it due to a mix of both technical and political reasons. Discussions on the NHIS were initiated again in 2007 and a draft health financing strategy was developed in 2009 to guide the country towards UHC. The strategy has not been finalized, implementation of UHC reforms has been relatively slow and it remains uncertain when the country will adopt a NHIS.

As the country continues to search for a UHC solution, a major concern is whether the NHIS is the most appropriate financing mechanism for Kenya, how such a mechanism should be designed and how to make it acceptable to stakeholders. Since stakeholders are key in any policy change; their preferences and interests can hinder policy formulation and implementation. A better understanding of stakeholders’ views and opinions regarding the NHIS design is important for future implementation. This paper traces the historical process of the development of the NHIS proposal and illuminates factors that led to the failure of implementing the policy using policy analysis techniques. Stakeholder’s views on the design are documented and lessons drawn to inform future development of health financing policies in Kenya.

## Methods

We conducted a retrospective policy analysis of the development of NHIS policy in Kenya. We assess actor’s perception on the NHIS design and examine factors leading to failure to implement the proposed reform strategy.

### Data collection

Data were obtained from various document reviews using a template which summarized details of the government documents, content and relevance, goals and objectives of the NHIS, key design features and policy development process. The review also analyzed type and roles of actors involved in the design. The key outputs of document review were organized into: documents reviewed and their relevance, historical context of the policy development, list of actors and their roles and implementation milestones. Documents reviewed are presented in Table 1.

**Table 1:** **List of documents reviewed**

**National reports and strategic proposals**	
	NSHI strategy: Comments and Suggestions of the Joint WHO/GTZ mission on Social Health Insurance in Kenya (1^st^ Mission) WHO/GTZ, June, 2003
	NSHI strategy: Comments and Suggestions of the Joint WHO/GTZ mission on Social Health Insurance in Kenya (2^nd^ mission) WHO/GTZ August, 2003
	NSHI strategy: Key findings and prerequisites for implementation (3^rd^ mission) WHO/GTZ/KfW Germany December, 2003
	NSHI strategy: Progress review and recommendations (4^th^ Mission) WHO/GTZ/DFID January 2004
	Progress review of the initial implementation stage and recommendations (5^th^ mission) Joint WHO – GTZ – DFID – ILO Mission to Kenya 29th March to 2nd April 2004
	Sessional paper No 2 on National Social Health Insurance in Kenya in Kenya May 2004
	The National Social Health Insurance Fund Bill, 2004
	Presentation by Amit Thakker CEO 2004. Avenue group in the informal session with stakeholders
	Financial projections and future bilateral/multilateral cooperation (6^th^ Mission) WHO/GTZ mission_21^st^ to 26^th^ June, 2004
	Carrin et al 2007 health financing reform in Kenya-assessing the social health insurance proposal
	IPAR 2005, Social Health Insurance Scheme for All Kenyans: Opportunities and Sustainability Potential IPAR policy Brief Volume11, Issue 2, 2005
	WHO 2006: Health financing reform in Kenya: assessing the social health insurance proposal
	MOPHS & MOMS March 2009, Towards a Health Financing Strategy for Kenya
	Nzoya Munguti, 2006, Review of Social Health Insurance in Kenya November 2006
**Documents from International meetings**	
	Opening of the International GTZ-ILO-WHO Conference on Social Health Insurance in Developing Countries Berlin, 5, 2005
	Social health insurance and its role in economic development and poverty reduction Inaugural Address by Mr. David Fuentes-Montero, Minister of Finance from the Republic of Costa Rica, in Central America
	The inclusion of the poor in social health insurance framework: the strategies applied in viet nam *By Dr Tran Van Tien during* The international Conference on Social Health Insurance in Developing Countries Berlin 5*;*– 7 December, 2005
	International Conference on Social Health Insurance in Developing Countries by Julio Frenk et al, 2005
	GTZ-ILO–WHO International Conference “Social Health Insurance in Developing Countries” Trends in health sector reform in Latin America in the 90’s and challenges for social protection in health in the 21st century by Eduardo Levcovitz
	Social health insurance: Social security and HIV/AIDS The experience of the National Social Security Fund by David Lambert Tumwesigye, Lusaka, Zambia, 9-12 August 2005
**Newspaper cuttings**	
	Health Matters: Plan to increase deductions by insurer continue to draw anger from workers: Daily Nation 17^th^ Aug, 2010 pg 11: “Civil servants reject NHIF dues
	Growing pains: Even as Government improves healthcare, questions emerge over financing Daily nation 17^th^ Aug, 2010 smart company pg 10: “The poor will finance the rich in the new medical plan
	Medical Care: Sunday nation 20^th^ June 2010 pg 4: hospital fund contribution to rise by 600 p.c
	Healthcare; Insured workers feel the pain as high premiums end up on payslips Daily nation 27^th^ July 2010 smart company pg 10: “Rising medical bill a bitter pill for employers
	Advertising feature: Daily Nation 27^th^ July 2010 pg 36: “The Evolution of NHIF”
	Medical fees row; setback for national health insurer: Daily nation 11^th^ Aug, 2010: Bid to block new NHIF rates certified ‘urgent

The gaps identified from the review were clarified during in-depth interviews conducted with seven different stakeholders in 2010. In-depth interviews provided an opportunity for detailed understanding of actor’s role in the policy making process, their perceptions of design and factors that may have necessitated failure to implement the policy. Some actors were no longer based in the participating institutions, in which case efforts were made to contact them because they were in a better position to discuss the study issues. Stakeholders were drawn from organizations including economists in the Ministries of Finance and Health, World Health Organization (WHO) country office, private health insurance companies, representatives from an umbrella body of Non-government organizations, and representative from development partners.

The topics covered included opinions on the NHIS policy process; health financing context; and stakeholders engaged in health care financing. Also explored were stakeholder’s perceptions on the health insurance design. Finally, views were sought on the actor’s opinions on the historical process of the NHIS policy development and the factors that led to the failed implementation. Where actors’ opinions differed with data reviewed from documents, clarifications were sought.

In-depth interviews were recorded (where consent was given) and detailed notes taken for those individuals who did not consent to recording. All interviews were transcribed and typed into Microsoft Word software. Informal analysis was conducted and summaries of the collected data made after each session for clarification or follow up. The data were stored and managed using QSR Nvivo 10 Software (© QSR international Pty 2007, Australia).

### Data analysis

A list of themes was developed from the desk review and later a complete thematic framework was developed as transcripts were examined. Analysis charts were then developed based on the policy analysis framework. Final analysis was organized around a description of the historical account on the development of the NHIS, role of actors and their influences, factors that led to the failed implementation.

Ethical approvals were granted by the Kenya Medical Research Institute (KEMRI) Ethics and Research Committee (Protocol number 1609). Written informed consent was obtained from all the interviewees. To maintain anonymity in reporting a set of broad actor groups are used to indicate the perspective of the information without linking to a particular actor.

## Results

### Historical account of NHIS in Kenya

A number of financing policies have been developed to guide the provision of effective and accessible healthcare based on the foundations of solidarity, responsibility, equity and transparency. Table 2 outlines the milestones towards the development of a NHIS in Kenya. Between 1965 and 1989 health care was financed through taxation in line with its policy of free medical care as envisaged in the Sessional paper 1 [15]. However, health care financing policies failed to enable Kenyans to access quality and affordable health care:

“*We realized that people are not accessing health, because of financial barriers. And health is putting about 1.5% of households below the poverty line. So we asked ourselves, ‘what is the best way forward?’ and in time to answer that question, we thought the best approach is for us to develop a financing strategy whose primary focus would be to tap the high out-of-pocket expenditure in an organized prepayment arrangement. That is how the debate of social health insurance came in and we prepared the sessional paper” (MoH actor).*

**Table 2:** **Key milestones towards NHI in Kenya**

**Period**	**key activities/outputs**
1965	✓ Sessional paper no 10 on “African Socialism and its application in Kenya” which outlined plans to provide welfare on a large scale. Government waived KES 5.00 charged to people
1966	✓ Creation of the NHIF through an act of parliament to replace the existing racially discriminative scheme by providing a contributory hospital based cover for all Kenyans aged over 18 years in formal employment and earning over Kshs 1,000.
1970	✓ Failure of local services to offer satisfactory services led to transfer of services to central government but no extra fund to meet the extra costs
1972	✓ Voluntary NHIF membership was introduced to bring on board the informal sector and those earning less than Kshs 1,000.
1986	✓ Sessional paper No. 1 of 1986 on “Economic Management for renewed growth”. Outlining Government priorities relating to financing of health care services including strengthening of NHIF and introduction of cost sharing in public health facilities.
1989	✓ The cost sharing policy introduced in the public health sector (user fees) to mobilize additional resources in the health sector, reduce excessive use of resources and improve the functioning of the referral system
1990	✓ Review of NHIF contribution premium rates having stagnated at Kshs 20 since inception of the fund to offset the impact of inflation and raising health care costs and also to generate additional resources towards financing of health care services
	✓ temporary suspension of user fees
1992	✓ Reintroduction of modest user fees
1994	✓ Cabinet approved the Kenya Health Policy Framework a blue print for priorities in health care
	✓Review of the NHIF from being a Government department within the ministry of health into a state corporation, through Act of parliament
November 2001	✓ First national congress on quality improvement in health medical research and traditional medicine
	✓ President directed ministers to take action on measures to establish a mandatory NSHI for all Kenyans
	✓ delegates adopted a resolution to include “right to health in the constitution” and adopted a task force report on affordable health care which recommended the establishment of NSHIF
January 2002	✓ Cabinet adopted a resolution for the establishment of NSHIF
May 2002	✓ Minister of Health established an inter sectoral task force to prepare a national strategy and Draft bill expected to lead to the establishment of NSHIF with its members from government and private sector
	✓ A task force completed its work and submitted a strategy report and a bill to the minister
2003	✓ Economic Recovery Strategy (ERS) for Wealth and Employment Creation (2003–2007) strategy aimed at transformation the existing NHIF into a NSHIF
June 2003	✓ MoH approached GTZ/WHO for technical support with 6th expert mission form June 2003-June 2004 to support implementation of the scheme once passed by law
June 2003	✓ 1st Technical mission which reviewed strategy and draft bill which led to the draft sessional paper no 2 of 2004
August 2003	✓ 2nd mission which focused on the legal aspects of the NSHI bill-benefit package, provider systems, transition of NHIF-NSHIF
October 2003	✓ 3rd Mission that focused on the health insurance management and financial feasibility of the implementation
January 2004	✓ 4th mission which focused on the progress towards implementation change management process and implementation of the working group
April 2004	✓ 5th Mission which reviewed progress to formulate mile stones
June 2004	✓ 6th Mission on financial projections and trained Kenyans on financial simulations tool
9th Dec 2004	✓ Debates in parliament and a bill was passed in but was not assented by the president on 31st Dec 2004

The proposed health insurance reforms were contained in the Sessional paper No. 2 of 2004 on the NHIS in Kenya [15]. The reforms sought to transform the NHIF into an NHIS. The genesis of a NHIS system was hatched in 2001. A taskforce was formed and consultations held in 15 districts across Kenya whose report recommended an NHIS [16]. In May 2002, an inter-sectoral task force was established to prepare a national strategy and legislation of Kenya’s NHIS. In June 2003, the MoH approached GTZ and WHO for technical support on setting up of a NHIS. Subsequent to the inter-sectoral task force report, six expert missions were set up to support the process [17].

The expert missions met between June 2003 to June 2004 and were responsible for various activities [16]:

- The 1^st^ mission conducted in June 2003 was responsible for reviewing the NHIS strategy paper, drafting the bill and the Sessional Paper number 2 on NHIS. Key recommendations raised by this mission was the need for a proper costing of the benefit package; having registration fees structured by levels of care to prevent overutilization; initial exclusion of long term illness; cost containment through quality management; mortuary charges limited to three days; and special review procedures for expensive drugs.
- The second technical mission set up in August 2003 focused on the legal aspects of the NHIS Bill, the benefit package and provider payments, the transition of the current NHIF into the NHIS and the implementation tasks. Key recommendations from this technical mission was the need to improve the NHIF image, which had a history of inefficiency and corruption, by promoting transparency and reducing the administrative costs to 10% for the first four years.
- The third Technical mission (October- November 2003) focused largely on the financial feasibility of the NHIS implementation. One key suggestion for this mission was inclusion of health facility based preventive services and curative care for both inpatient and outpatient services. HIV/AIDS and Tuberculosis were to be included but accounted for separately.
- The fourth mission set up in January 2004 concentrated on progress toward implementation of the NHIS, addressing the change management process and the activities of the NHIF working groups. It reviewed progress towards implementation of the NHIS, drafted the final version of the bill, and explored opportunities of integrating retirement schemes of armed forces later.
- The fifth Technical mission conducted between March and April 2004 aimed at reviewing the progress on the initial stage of NHIS implementation and to formulate milestones in the full implementation.
- The final Technical mission was organized in June 2004. It provided the revised financial projections through informal discussions with stakeholders and parliamentary committee on health. The main set back was the announcement of the introduction of free health care services for all Kenyans with effect from 1st July 2004. This reduced the willingness of the informal sector to contribute to the scheme.

In June 2004 the NHIS bill was tabled in parliament. The bill sought to establish a mandatory NHIS by July 2004 but the president declined to assent the bill citing problems related to technical design, affordability, implementation and sustainability. The bill was returned to parliament with a memo on the proposed changes¸ but these were never addressed. Efforts were made to establish another committee to spearhead the process afresh in 2007. However, since the previous attempt had polarized key stakeholders, the committee decided to use the national development blue print as the vehicle for the health care financing agenda.

“*I think the year was to give us a break. Because when you push something too far, and you do not succeed, you do not want to start the whole thing too fast before your enemies have probably disbanded. We saw that we require one year, for us to rethink the whole process and probably use another platform to move the same agenda. And in this case we were just developing the vision 2030. And one of the pillars of vision 2030 is having a social health insurance framework. So we wanted to use the vision 2030, because remember the initial entry was the economic recovery strategy, we failed” (MoH actor).*

Overall, the development of NHIS policy was anchored on the national development blue print platform which all government agenda are based on. Despite this, there has been limited success towards realizing the NHIS vision.

### Stakeholder’s perceptions of the NHIS design

#### Contribution methods

The proposed sources of financing for the NHIS was the government through government revenue and earmarked taxes, the employed (formal sector workers) through payroll harmonization, contributions of employers and the self-employed as well as donations and grants. Employees and employers were to contribute on an income-rated basis while the self-employed were to contribute an affordable flat rate. In essence, those working in the formal sector and their employers were expected to contribute more than the self-employed. The government was then expected to contribute for the poor from other sources such as donations and grants.

Collection of contributions in the formal sector was based on an assumption that all employers will comply with the obligation to pay their contributions. Measures to improve compliance were discussed, based on improving the process of information exchange with business-registering authorities for registration, and with tax revenue authorities for contribution collection. For the informal sector population, contributions were to be collected by various organizations close to the population. These organizations include cooperatives, welfare organizations, trade associations and churches. Organizations were to be contracted for this purpose and remunerated for collection that they deliver. In addition, some of these organizations were to be licensed to issue or stamp the social health insurance card.

### Pooling of revenue

There were varied opinions among actors as to whether the best option was to have single or multiple pools. The 2004 proposal was to re-organize the NHIF as the main vehicle for the fund rather than setting up a new system. The proposal drew negative reactions as NHIF had several transparency challenges including its administrative costs. However, most actors noted that it was the only sensible thing to do then. Arguments against multiple pools revolved around efficiency and cost of running the system, while others argued that

“*Multiple pools break the solidarity especially in a small system. For a small country, you need a strong solidarity element and once you categorize people, you will have to have a lot of cross subsidies across different groups” (Donor actor).*

There were arguments that a single pool has the administrative and logistical problems associated with monopoly which are linked to inefficiencies if not well designed. Most of the inefficiencies of the NHIF were associated with the monopolistic market and there were fears that the same weaknesses would exist if the NHIS did not incorporate some elements of competition.

### Purchasing

There was an agreement that purchasing health care through a common pool was a good idea:

“w*e would want all Kenyans to pull resources into one pot and use it to finance the health care, for both the rich and the poor” (MoH Actor).*

However*,* the contentious issues revolved around whether to have single or multiple purchasers of which the latter would allow competition:

“*There were some people who could not understand the request from the private sector, so they ended up signing an agreement to allow competition for NHIF only to realize the implications later” (Treasury actor).*

Most players agreed that although the vehicle to implement the scheme was NHIF, this ought to have waited until it was restructured:

“*NHIF cannot be the same person who is collecting the money and at the same time utilizing that money” (NGO Actor).*

Some even suggested the need to separate revenue collection and purchasing functions.

Overall, there appeared to be divergent views on the role of purchasers and providers. Some suggested a separate body to collect the money and administer it while a separate body is responsible for purchasing. Separating these roles is critical for transparency and efficiency. There was general agreement that purchasers-provider arrangement should be accompanied by effective regulation and enforcement:

“:*…because you realize then you would have about 300 purchasers …. And if they are all doing whatever they want to do, it will be total chaos; some will be efficient while others will not” MoH Actor.*

### Provider payment mechanisms

In terms of provider payment, the design proposed a flat remuneration rate per inpatient day. For outpatient care, a flat fee per visit (case payment) was to be paid to providers. Maximum provider payment levels and special approval for treatment abroad were also suggested to help contain costs. The exact remuneration levels were not finalized but were suggested to be KES 1500–2500 per inpatient day and KES 100–400 per outpatient visit, based on selected health facilities. These rates were based on the assessment of the financial needs reported by Mission and Government Hospitals with an extra allowance for higher quality services and infrastructure development. Reductions in fee levels were considered in the short run if health facilities cannot provide the full benefit package.

### Accreditation of health care providers

Different stakeholders recommended that the design should have a separate and autonomous department to conduct accreditation that is independent of the purchaser. That way the primary responsibility of the MoH will be policy making and divorces itself from service provision and accreditation. Most stakeholders agreed that there needs to be a clear set of criteria for accrediting facilities through some form of quasi government organization that is independent of the revenue collection and purchasing agencies.

### Benefit package

The proposed bill generally provided for one minimum basic package. The package was to cover both outpatient and inpatient services such as medical consultation, specialist care, drugs hospitalisation, dental care, referral and specialised treatment and other benefits as the board may approve. The main contentious issues were uncertainty of how HIV and specialised care would be provided. There were views that there were no actuarial studies done questioning the sustainability of the scheme:

“....*When they talk about management of HIV, there are so many ways of managing HIV/AIDS. So when they say renal management, there are so many elements of renal management, there is the dialysis and all that. And one episode of dialysis can cost you a substantial amount of money. But then they say they can afford that there is still some doubt. And then we have been asking for some evidence on the actuarial studies that has informed that benefit package” (private sector actor).*

However actors generally agreed that there needed to be a minimum package excluding expensive medical treatments:

“*And I think the argument then was that, let’s cluster this thing into three blocks. One is the primary healthcare. Things which are social… you know public goods in nature, and things where the private sector will not be interested to come in. and you say those things will be purchased and paid for by the government; immunization, condoms, you do not want the private sector to come in just because in the event that they don’t provide the goods, then the repercussions will be more than if the government provided them. Then we look at the secondary level” (MoH –actor).*

### Factors influencing the realization of the bill

#### Cost of implementation

One concern raised as the reason behind failure for the president to assent to the bill was based on the understanding of the implementation, affordability and its implication to the economy. The proposed roll out of the design in phases was not clearly communicated to key players. Stakeholders in the finance ministry perceived that the cost of implementing the scheme was high with implications to the economy:

“*…because they looked at the cost of the entire project and said it was very expensive, but what they failed to flag out, is the process of implementing it in stages… which was part of the design…. so what treasury did is to flag out the final cost of the project, which was coming to around forty billion Kenya shillings, and said this is too expensive” (MoH actor).*

On the other hand, there was a perception that some government departments deliberately painted the scheme as expensive to generate apathy among major development agencies*:*

*“it was almost impossible to know the financial implications upfront” (Development partner).*

The cost of care was also not perceived to be commensurate to the quality of service provided in government facilities.

#### Inadequate communication strategies

A dominating theme was that of inadequate understanding of the design and how the policy was to be implemented. This generated misconceptions around the purpose of the bill and timing as some actors perceived this as an avenue to provide political mileage to others. Underlying these issues was inadequate communication strategy to articulate the design, process and the economic implications adequately leading to various “versions of the design” to the public as was described:

*“The only issue is that I blame the political system that was supposed to connect; the technical people and the public. This failed us. They didn’t pass the correct message to the public. You can’t go to Kenyans and tell them we are going to consume … free healthcare services. There is always a price for everything. So that is one area we failed” (MoH actor).*

In essence information on the design was “sparingly provided” leading to patches of the facts that appeared to generate “uninformed discussions” thought of as deliberate attempts to conceal realities of the policy:

“…..*I would say there was some element of mischief…(MoH actor).*

Lack of a guided communication strategy generated a vacuum of factual information flow to various players partly due to complacency in the part of the government to take leadership as well as various players within and outside government departments opposing the bill covertly as one actor pointed out;

“*I can tell you there were people silently… expressing some discontent about the draft proposal...... If you are to compare it to some strategies that we have from other countries, you will realize that ours does not bring out the issues clearly, like the role of the different organs like NHIF, the role of the private sector and all that” (MoH Actor).*

The public was not enlightened on the key concerns while the positive aspects were watered down on the basis of feasibility and cost of implementation.

#### Fear of implications of the changes

The fear of the implications of changes among the private sector on their business threatened support and success that had been gained up to the passing of the bill. The immediate response of private sector actors was the desire to maintain the status quo:

“*We have since come through a very big transformation….what will it mean to our businesses? So it was a genuine concern” (private sector actor).*

The underlying issue was loss of revenue through outpatient payments made to private health insurance. The suspicions was based on uncertainty on what the bill would bring, fuelling and cementing the positions of the two opposing camps. The dissenting voices defeated even those in the private sector who were pro the changes:

“*They did not understand how this is going to work out. We were not seeing each other as players and all of us have space. … if this system works out then it means I will lose my…cut!……I will reduce the profits that I get!” (NGO actor).*

#### Trust, transparency and governance

Underlying the fear described above was mistrust associated with perceived lack of government’s commitment to instil transparency and good governance. The perceived mistrust affected active engagement of all key players dampening the spirit of collective bargain. Minimal involvement of the private sector players was interpreted as lack of transparency hampering the process of support. Actors contended that it was important to consider the role of private sector in health care delivery and financing:

*“other non-government players are very strong. So if you don’t have their backing whether your idea was good or not it’s likely to fail purely because you did not involve, it was not participatory, you see?” (Private sector actor).*

Lack of trust on government’s leadership by various private sector players bred opposition. A number of reports on lack of transparency on the use of NHIF fund further perpetuated the mistrust and eroded the confidence that had been gained.

“*And at that time the NHIF was very inefficient, spending too much money on administration, and we thought in that Bill we should cut the level of administration costs, and we put it at 10%. And we also wanted to look into contribution at a rate of 2.5% per person. So that was not very contentious but the most contentious issue was this animal we are giving money, how efficient is the animal that is now NHIF” (Private sector actor).*

The net effect was a blame game among different actors hiding under “lack of involvement” of a broader set of key players. Actors expressed mixed feelings about the engagement with the MoH with some reporting involvement throughout the process:

“*And during the preparation, we mobilized all the stakeholders; Central Organization of Trade Union (COTU), Federation of Kenya Employers (FKE), private sector, teachers union… to rally them behind …… what we thought was a noble objective” (MoH actor).*

The private sector actors on the other side reported scanty initial engagement, although there were efforts to engage more players post design stage.

Transparency and trust were factors that were linked to use of public funds collected given the history of NHIF. Real or perceived mismanagement of funds generated debate on the potential success of the program. Historic experiences and governance context limited acceptability of design: “W*e have given you taxes, since we got independence. Can we see that you have taken care of the assets that we have given you?” (private sector actor).*

#### International influence

Initially there was support from international actors indicated by their involvement at the design stage. However, some actors perceived a clash of their role and that of international programs based on vertical approaches inherent in the health system. On one hand it was perceived that donors had different priorities that did not match those of government and that they preferred funding parallel programs with limited integration if any:

“….*you find people concentrating on certain things that are not the priority of the country.. but these guys don’t want to hear about health care financing broadly they are pouring a lot of money may be to HIV” ( Treasury-actor).*

*“Those vertical programs have an impact but I don’t think it’s a real impact … I have a feeling that it’s very program oriented, we want to do this, once we are done, that’s it (Health insurance actor).*

Such perceptions, led to doubts over the government’s ability to finance the project in the absence of donor funding. Secondly, there was fear among donors that they may also lose business in various projects once a comprehensive health care financing strategy is in place:

“T*here were two issues from the donors. One of the issues was that ‘if this thing happens, what will be our role in the health sector? That if the MoH is able to mobilize ninety billion, and their money (donor funding) then was about a billion, you know, they have no role in the health sector. So that was a major fear, and in fact they used that fear to cause some key donors to agitate the private sector to campaign against the social health (private sector actor).*

Donors were also uncomfortable with the issue of access of services to the poor and how the government will ensure equity issues are covered. In addition, the design was criticized for not meeting the international standards when compared with strategies from other countries. The bill did not articulate the design issues clearly.

## Discussion

This paper aimed to provide a historical account of the development of a NHIS policy in Kenya, illuminate factors that led to the failure of its implementation using retrospective policy analysis, and draw lessons for future policy design. In the last two decades a number of countries have implemented NHIS with varying outcomes, or are in the process of doing so. Rwanda, Burkina Faso, Kenya, Nigeria, and South Africa have either started a national health insurance program or are in the advanced stages of starting one [18]. Ghana adopted the NHIS in 2003, which was fully implemented two years later [19], with relative success in enrollment, utilization, increasing access to formal services [20,21]. The unique position of Kenya having not implemented the proposed design nearly ten years later can help draw out key lessons for countries which are thinking of formulating NHIS.

First, the consultative process of developing the policy through expert missions was valuable as it drew from each other’s recommendations and defined the subsequent plan of action. However it lacked a comprehensive communication strategy to manage the expectations and reactions of key stakeholders such as organised employer organisations and private sector. Future reform drivers may consider developing a stakeholder management strategy that will provide information to key players during the design period. As was observed in the Tanzania and South African experience, stakeholder management should target actors who are pro-policy and actively engage those against it [22].

This observation brings to fore a number of lessons for future reform process. Frist, understanding actor’s interests on specific design elements may facilitate implementation. For example, key interests instrumental in driving covert opposition by different players was lack of accountability as trails of mistrust by key players was widespread, a perception fuelled by failure of government to account for the funds collected through NHIF. This led to the private sector players being opposed to collecting revenue through a public sector authority that pools revenue to a central fund administered by a government-controlled body. In retaliation the private sector proposed the need for competition as critical element in ensuring transparency and inculcation of spirit of ownership. The second interest was the profit motive that was threatened by a wider scheme that would ‘eat’ into actors’ potential “business space”. The need for the actor’s understanding of specific elements of the policy is reflected in the stakeholders’ understanding of the one-time premium policy in Ghana which led to several misinterpretations [23].

The issue of trust brings in the second lesson where future proposals should develop a continuous process of cultivating the spirit of trust over time through governance structures that promote accountability. Such structures will attract contributions towards sustainability. For consumers to enroll in health insurance, they should trust that insurers use their funds to reimburse providers who will deliver quality care when needed [24]. Investing in administrative efficiency and transparency such as use of efficient electronic system of payment may facilitate trust. Having proper accountability channels include mechanisms for members to raise complaints related to the insurer is also critical. There may be need to rebrand institutions such as NHIF to enhance trust among contributors. We are cognizant that building trust takes time but investing in efficient and transparent systems and effective engagement of stakeholders throughout the design process is important for acceptability. Development partners can play an important role in supporting restructuring of key institutions to act as drivers for change. In addition, the widespread perception of poor quality of services in public health systems, may limit utilization of services regardless of whether the NHIF is restructured and a UHC policy put in place. Trust in the public health system and the government’s ability to provide services may frustrate any meaningful reforms. Such opposition may be offset by building efficient service delivery structures that are responsive to population needs. Improving service delivery at all levels of care is a prerequisite for making progress towards UHC. Experiences from Rwanda showed that, managers, providers and policymakers need to think about a wide range of initiatives that enhance trust and caring, and to design trust building structures and practices in the consumer–insurance–provider arrangement [24].

The third lesson is around active sensitization and engagement of key players through a well-organized leadership. Public engagement and encouraging public dialogue on key issues from the early design stage is key to success. Although there were divergent views on the nature of stakeholder engagement during the design stage, it was clear that the covert opposition due to varied interest negated the gains made. Engaging the private sector as a major stakeholder is critical to allow deliberations and dialogue, while maintaining leadership and managing conflicts at the design stage due to differing interests.

The South African experience is an example of how stakeholders can derail implementation. Private sector companies and the divergent views of the governing party-aligned trade unions that supported a reform which collects revenue through a public sector authority and pools this revenue in a central fund administered by a government-controlled body, were opposed by those who wanted a managed competition [22].

The fourth lesson is the need to assess specific design barriers. For example, actors had concerns on some elements of the design that were linked to cost cutting measures, efficiency and logistical process of implementation. Confusion with concepts used in universal coverage reforms were also reported in South Africa, with the term NHIS being used in their health care financing reforms for a system that has to be largely tax-funded [25]. Finally, future reforms process should focus on how to garner support from large employers by persuading them that the proposals would reduce their workforce costs. The win-win situation is critical for future implementation.

This study had a number of limitations. The study is limited by its retrospective nature which may not capture all relevant issues. In addition, the views may not be universal of all stakeholders since we could not interview the entire set of actors involved as some of them had left their previous positions and were not willing to participate. Thirdly, the study only focuses on the policy design and formulation phase which limits us from drawing lessons from literature as most studies assess the effect of implementing NHIS. Few have examined stakeholder perceptions of the design at inception such as those from Ghana which focus on challenges of implementation process [26], while studies from Nigeria and Ghana, show the effect of NHIS on health indicators [27].

## Conclusion

The Kenyan experience shows that the thrust towards UHC over the last decade was affected by the complex interaction of actors’ understanding of the design, inadequate management of the process and the covert opposition of actors in both government and private sectors whose interests were not catered for. Future reforms in health care financing towards UHC in Kenya, should not only focus on the design of a viable national health insurance but also devise ways of building trust to existing health care systems, the public and institutions mandated to provide leadership in the reform process.

## Abbreviations

UHC: Universal Health Coverage
NHI: National Health Insurance
LMICs: Low-and -Middle Income Countries
OOP: Out-of-pocket
NHIS: National health insurance scheme
NHIF: National Hospital Insurance Fund
KEMRI: Kenya Medical Research Institute
WHO: World Health Organization
FKE: Federation of Kenya Employers
COTU: Central Organization of Trade Union
